# Comparison of neonatal red cell transfusion reporting in neonatal intensive care units with blood product issue data: a validation study

**DOI:** 10.1186/s12887-018-1005-2

**Published:** 2018-02-23

**Authors:** Jillian A. Patterson, Jennifer R. Bowen, Sally Francis, Jane B. Ford

**Affiliations:** 1 Clinical and Population Perinatal Health Research, Kolling Institute, Northern Sydney Local Health District, St Leonards, NSW 2065 Australia; 2 Sydney Medical School Northern, University of Sydney, Sydney, Australia; 3 Royal North Shore Hospital, Northern Sydney Local Health District, St Leonards, NSW 2065 Australia; 4 BloodWatch Program, NSW Clinical Excellence Commission, Sydney, NSW 2000 Australia

**Keywords:** Sensitivity, Neonatal intensive care, Transfusion, Validity

## Abstract

**Background:**

Infants in Neonatal Intensive Care Units represent a heavily transfused population, and are the focus of much research interest. Such research commonly relies on custom research databases or routinely collected data. Knowledge of the accuracy of transfusion recording in these databases is important. This study aims to assess the reporting of red blood cell transfusion neonatal intensive care unit data compared with routinely collected hospital blood bank (“Blood Watch”) data.

**Methods:**

Blood Watch data was linked with the NICUS Data Collection, and with routinely collected birth and hospital data for births between 2007 and 2010. The sensitivity, specificity, and positive and negative predictive values for transfusion were calculated, compared to the Blood Watch data. The agreement between the NICUS and Blood Watch datasets on quantity transfused was also assessed.

**Results:**

Data was available on 3934 infants, of which 16.2% were transfused. Transfusion was reported in the NICUS Data Collection with high specificity (98.3%, 95% confidence interval (97.8%,98.7%)), but with some under-enumeration (sensitivity 89.2% (95% CI 86.5%,91.5%)). There was excellent agreement between the NICUS and Blood Watch datasets on quantity transfused (Kappa 0.90, 95% CI (0.88,0.92)). Transfusion reporting in the hospital data for these infants was also reliably reported (Sensitivity 83.7% (95% CI 80.6%,86.5%), specificity 99.1% (95% CI 98.7%,99.4%)).

**Conclusions:**

Transfusion is reliably reported in the neonatal intensive care unit data, with some under-reporting, and quantity transfused is well recorded. The NICUS Data Collection provides useful information on blood transfusions, including quantity of blood transfused in a high risk population.

**Electronic supplementary material:**

The online version of this article (10.1186/s12887-018-1005-2) contains supplementary material, which is available to authorized users.

## Background

Infants, particularly low birth weight and premature neonates represent a highly transfused population [1–3], with many of these transfusions occurring within neonatal intensive care units (NICU) [1]. Understanding the frequency of, and reasons and risk factors for transfusion in this population is important for service delivery, and to monitor the appropriateness of transfusion in these infants. The data needed to conduct such research are commonly sourced from medical record review [4, 5], routinely collected administrative databases [1], or custom research datasets [3, 6, 7]. It is important to know how accurately transfusion information is recorded in these databases when using any of these data sources for research. As these data sources are frequently collected for purposes other than clinical management, the reliability of the information they contain is unknown.

Where a high level of inaccuracies are present in the data it can affect research findings based on the data. In databases where transfusions are frequently recorded when they are not actually given (low sensitivity) or if a large proportion of transfusions are not recorded for patients receiving them (low specificity) then risk estimates based on these data can be biased or diluted. This problem is exacerbated where the likelihood of a transfusion being recorded varies over time or according to the severity of the patient’s condition. Comparison of transfusion reporting across databases allows the extent of over- and under-reporting to be estimated. In New South Wales (NSW), Australia, one custom research database on ill neonates is the Neonatal Intensive Care Units (NICUS) Data Collection. This data collection contains information abstracted from the medical record on selected infants admitted to NICU [8]. In 2007, information on red blood cell transfusion was included in this database for the first time, but the accuracy of the data collected has not been assessed. In this time period, hospital blood banks have been submitting information to a central database ‘Blood Watch’ on each red cell unit issued. Linkage of these databases, along with routinely collected hospital admission and birth data, provides the opportunity to assess the reporting of transfusion in the NICUS and hospital data.

Previous studies in general inpatient populations, and in obstetric populations have found that transfusion is reported with high specificity [9], but some under-enumeration [10, 11]. These studies have been based on routinely collected hospital data, which records only fact of transfusion, rather than quantity transfused. Quantity transfused is often an indicator of the severity of the condition of the infant. Both the NICUS and Blood Watch Data Collections contain additional information on the quantity of blood transfused, which could expand the scope of future studies on transfusion in this population. This study aims to identify the accuracy of using neonatal intensive care unit and hospital data to identify neonatal transfusions among babies admitted to neonatal intensive care.

## Methods

The study population was all liveborn infants of at least 23 weeks gestation born in one of six NSW hospitals with Neonatal Intensive Care Unit (NICU) facilities and maternity services who submitted data to the Blood Watch database (two additional hospitals with NICU facilities do not have maternity services, and one did not have complete data available). Infants were eligible if they were admitted to NICU and had data available across the Perinatal, Admitted Patients and Neonatal Intensive Care Surveillance datasets. Only records pertaining to the hospital of birth were included; transfusions occurring following transfer to another hospital were not examined. Births were identified from the Perinatal Data Collection, a statutory collection of all births in NSW. Data on transfusion was available from three sources: the Clinical Excellence Commission ‘Blood Watch’ data, the Admitted Patient Data Collection (‘hospital data’), and the Neonatal Intensive Care Units (NICUS) Data Collection. Data was available from 1 January 2007 to 31 December 2010.

The Blood Watch data incorporates information from the Australian Red Cross Blood Service on the type and number of products issued to a patient, and combines this with pathology test results available for the patient. This data is submitted electronically to a central database by laboratories in participating hospitals. Each unit of blood issued is an individual record in this data, allowing calculation of number of units transfused. Transfusion in this dataset was defined as the issue (without return) of one or more units of red cells to a patient. Standard issue red cell units and the smaller pedipacks (single unit split into 4) commonly used for infants and children are each counted as a single transfusion.

The Admitted Patient Data Collection is a census of admissions to NSW hospitals. Data on up to 50 diagnoses and procedures are collected and coded according to the International Classification of Diseases version 10, Australian Modification (ICD10-AM) and the Australian Classification of Health Interventions (ACHI) respectively. Transfusion was identified from a record of transfusion of red cells or exchange transfusion in the procedure codes. The number or volume of transfusions was not available from the hospital data. The Perinatal Data Collection was used to identify birth admissions (that is, the admission during which the delivery of the infant took place). The Perinatal Data Collection also provided information on the pregnancy and the birth of the infant.

Data on infants meeting one or more of the following criteria are recorded in the NICUS database: < 32 weeks gestation, birthweight ≤1500 g, requiring assisted ventilation, undergoing surgery requiring the opening of a body cavity, insertion of a central line or intentional hypothermia for infants with hypoxic ischaemic encephalopathy. From 1 January 2007, this database contains a record of each transfusion performed within the NICU in these infants. Not all infants admitted to NICU are eligible for inclusion in this database.

Probabilistic linkage was used to combine records from each database, belonging to the same infant, corresponding to their birth admission. Linkage was performed by the NSW Centre for Health Record Linkage using privacy preserving principles, with deidentified data provided to researchers.

Transfusion reported in the Blood Watch data was considered the “gold standard”. Sensitivity, specificity and positive and negative predictive values were calculated comparing reporting of ‘any red cell transfusion’ in Blood Watch and the NICUS Data Collection, and are reported with exact binomial confidence limits. Agreement between quantity transfused was assessed using weighted Kappa statistics for Blood Watch and NICUS data, where kappa values less than 0.4 represent poor agreement, 0.4–0.75 good agreement and greater than 0.75 represent excellent agreement beyond chance [12]. Due to small numbers of infants and the narrow range of number of transfusions given, number of transfusions was grouped into 1–2, 3–4, 5–9 and 10+ to reflect clinical severity. Trends in rates were assessed using the Cochran-Armitage test for trend.

A secondary analysis was conducted comparing reporting of transfusion in the hospital data (compared with Blood Watch data) among this high risk population.

All references to transfusion in this manuscript refer to red cell transfusions. All analysis was performed in SAS 9.3. This study received ethical approval from the NSW Population and Health Services Research Ethics Committee.

## Results

**Fig. 1:**
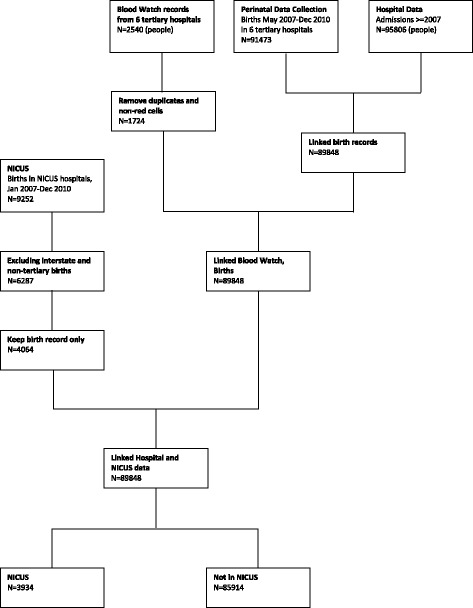
Identification of related records for infants registered in the Neonatal Intensive Care Units’ Data Collection

Between January 2007 and December 2010, there were 91,473 infants liveborn in tertiary hospitals. Of these 3934 (4.3%) had records across the birth, hospital and NICUS databases and were included for analysis (Fig. 1, also see Additional file 1: Table S1, which describes the characteristics of the population). Pre-transfusion haemoglobin measures were available for 505 (12.8%) infants, with the lowest recorded haemoglobin ranging from 39 g/L to 198 g/L (median 91 g/L). Almost half (1833, 46.6%) of NICU infants were ≥33 weeks gestation, 606 (15.4%) had a five minute Apgar score of < 7 and 979 (24.9%) were from multifetal pregnancies.

The Blood Watch data reported information for 637 (16.2%) transfused infants in the NICUS Data Collection, and NICUS had transfusion data for 628 (15.9%) (Table 1). There was no significant change over time in transfusion rates reported in any dataset (Blood Watch *p* = 0.08, NICUS data *p* = 0.14, Hospital *p* = 0.45) Red cell transfusion was reliably reported in the NICUS Data Collection, but with some false positives (Sensitivity 89.2% (95% CI 86.5%,91.5%), Specificity 98.3% (97.8%,98.7%), PPV 90.9 (88.3%,93.0%)) (Table 2). The sensitivity of transfusion reporting was higher in the earlier years, and the decline in sensitivity was not associated with any particular hospital.

**Table 1:** Transfusion reporting in Blood Watch (hospital blood bank) vs Neonatal Intensive Care Units’ (NICUS) Data Collection for infants registered in the NICUS Data Collection

		Blood Watch Data Collection	
		Transfusion	No Transfusion
NICUS Data Collection	Transfusion	568 (14.4)	57 (1.5)
	No Transfusion	69 (1.8)	3240 (82.4)

**Table 2:** Sensitivity and Specificity of transfusion reporting in Neonatal Intensive Care Units’ (NICUS) Data Collection compared with Blood Watch (hospital blood bank) data collection for infants in the NICUS Data Collection

		Blood Watch data	NICUS rate	Sensitivity	Specificity	Positive Predictive Value	Negative Predictive Value
Total		637/ 3934 (16.2)	625/ 3934 (15.9)	89.2 (86.5, 91.5)	98.3 (97.8, 98.7)	90.9 (88.3, 93.0)	97.9 (97.4, 98.4)
Year	2007	154/ 905 (17.0)	149/ 905 (16.5)	92.9 (87.6, 96.4)	99.2 (98.3, 99.7)	96.0 (91.4, 98.5)	98.5 (97.4, 99.3)
	2008	172/ 980 (17.6)	172/ 980 (17.6)	89.5 (84.0, 93.7)	97.8 (96.5, 98.7)	89.5 (84.0, 93.7)	97.8 (96.5, 98.7)
	2009	157/ 992 (15.8)	147/ 992 (14.8)	87.3 (81.0, 92.0)	98.8 (97.8, 99.4)	93.2 (87.8, 96.7)	97.6 (96.4, 98.5)
	2010	154/ 1057 (14.6)	157/ 1057 (14.9)	87.0 (80.7, 91.9)	97.5 (96.2, 98.4)	85.4 (78.8, 90.5)	97.8 (96.6, 98.6)

There was excellent agreement between the Blood Watch and NICUS databases in terms of quantity transfused (Weighted Kappa 0.90 (0.88,0.92)) (Table 3). The proportion of transfusions reported in the NICUS data increased as quantity transfused increased (Table 4), with all infants receiving transfusions of 10 or more units identified as transfused in the NICUS data. The proportion of 1 and 2 unit transfusions identified in the NICUS data was slightly better in earlier years, but the accuracy of the reporting of larger volume transfusions remained high across the study period.

**Table 3:** Agreement between Neonatal Intensive Care Units (NICUS) Data Collection and the Blood Watch (Hospital blood bank) Data Collection on quantity transfused for infants registered in the NICUS Data Collection

	Blood Watch				
NICUS Data Collection	0 units	1–2 units	3–4 units	5–9 units	10 plus units
None	3240	56	10	3	0
1–2 units	40	306	17	3	0
3–4 units	8	10	115	16	0
5–9 units	8	1	5	68	2
10 plus units	1	0	0	7	18
Weighted Kappa (95% CI)	0.90 (0.88, 0.92)	<.0001

**Table 4:** Proportion of Blood Watch (hospital blood bank) transfusions identified in the Neonatal Intensive Care Units’ (NICUS) Data Collection, by volume transfused

		Quantity transfused (units)	NICUS Data Collection (N)	Blood Watch Data (N)	% of transfusions identified in the NICUS Data Collection Row %
Total		1 to 2	316	372	84.9
		3 to 4	137	147	93.2
		5 to 9	94	97	96.9
		10 or more	20	20	100.0
Year	2007	1 to 2	81	89	91.0
		3 to 4	32	34	94.1
		5 to 9	23	24	95.8
		10 or more	7	7	100.0
	2008	1 to 2	87	100	87.0
		3 to 4	29	33	87.9
		5 to 9	32	33	97.0
		10 or more	6	6	100.0
	2009	1 to 2	75	92	81.5
		3 to 4	35	37	94.6
		5 to 9	23	24	95.8
		10 or more	4	4	100.0
	2010	1 to 2	73	91	80.2
		3 to 4	41	43	95.3
		5 to 9	16	16	100.0
		10 or more	3	3	100.0

Within this population of infants in NICU, the hospital data reported on 562 (14.3%) transfused infants. Compared with Blood Watch data, the reporting of transfusion in the hospital data for this subpopulation had sensitivity 83.7% (80.6%, 86.5%), specificity 99.1% (98.7%, 99.4%), and PPV 94.8% (92.7%, 96.5%) and showed a similar decline in accuracy of reporting over the study period to NICU data (PPV declined from 97.0% (92.5%, 99.2%) in 2007 to 90.5% (85.6%, 94.7%) in 2010) (See Appendix).

Of the 69 infants with transfusion reported in Blood Watch but not NICUS data (false negatives), 81% (*N* = 56) received 1–2 units of blood, 61% (*N* = 42) were ≤32 weeks gestation, and 20% (*N* = 14) were term infants. Of the 57 reported in NICUS but not in Blood watch (false positives), 70% received 1–2 units of blood, 54% (*N* = 31) were infants ≤32 weeks and 26% (*N* = 15) were term infants.

## Discussion

Between 2007 and 2010 data was available on 3934 infants admitted to a NICU and included in the NICUS database. Of these infants, around one in six received a red cell transfusion. Overall, there was good reporting of transfusion in neonatal intensive care unit data, but with some under-enumeration (sensitivity 89.2%). Transfusion was reported with high specificity (98.3%), meaning that cases reported are likely to be true cases. Although the NICUS database is well established (with reporting commencing in 1992), blood transfusion information has only been collected from 2007. A one off linkage with the Blood Watch data submitted by hospital blood banks provided an important opportunity to understand previously un-validated data items in NICUS and hospital data. Validating transfusion reporting via medical record review would be labour intensive and costly, and thus potentially difficult to justify, however through linkage of datasets reporting transfusion using different sources, such validation becomes more feasible.

This relatively high transfusion rate of infants admitted to a NICU reflects the more serious condition of these infants compared with the general neonatal population which has a transfusion rate of 5.4 per 1000 births [1]. It is possible that reporting in this high risk population is not generalizable to all infants, however most infants are transfused in the NICU setting [1]. For the same group of infants, transfusion reporting in the hospital data was less complete, with sensitivity 83.7% and specificity of 99.1%. This degree of ascertainment is consistent with the reporting of transfusion in adults [9] and in the obstetric population [11]. The NICUS data, being a more specialized collection, was better able to identify transfused infants compared with hospital data. This may be due to differences between NICUS and hospital records in how the data is collected. Hospital transfusion is coded from the medical record, and relies on documentation of transfusion in the notes, whereas NICUS data is collected by clinical nurse specialists [13] who have ICU experience, and may be more familiar with finding this information in the medical record. Nevertheless, the hospital data has reasonable reporting of transfusion and, assuming overall consistency of reporting, provides a valuable data source on transfusions outside of NICU (which represent at least 7% of transfusions) [1].

Excellent agreement was noted on quantity transfused between the Blood Watch and NICUS databases. Few databases collect quantity transfused, and so validation studies to date have examined only reporting of fact of transfusion [9–11]. Given the important additional detail provided by quantity transfused, it is reassuring to know that this information is reliably reported. Better reporting was observed in higher volume transfusions. This is consistent with the findings of other validation studies, where more serious conditions are more likely to be reported [11, 14–16].

Over time, the sensitivity of transfusion reporting declined in the NICUS data. This decline seemed to be largely among the smaller volume transfusions. The reasons for this decline are unclear, however a similar decline was not seen when comparing hospital and Blood Watch data reporting, suggesting there has been little change to the reporting in the gold standard.

In some cases, false positives and negatives will be due to clerical error, however some differences were identified between infants who were identified as having transfusion compared with those who were not. False positives and negatives in the data were most frequently low volume transfusions. In some cases this may reflect situations where the blood was ordered but not transfused and not returned to the blood bank or the transfusion was not noted in the medical record. This is also consistent with the finding of better reporting in more severe cases, where transfusion is expected, and where multiple transfusion occasions make it more likely that a case will be identified. Transfusions among term infants were overrepresented in the false negatives, suggesting that where transfusion is unexpected, coders may not specifically look for evidence of transfusion.

This study has a number of strengths. By obtaining transfusion information from independent sources, the reliability of transfusion reporting between the sources was able to be assessed. In particular, as both Blood Watch and NICUS databases provide data on quantity of blood transfused, the agreement in quantity transfused was able to be assessed. A potential limitation of the study is the narrow definition of the study population, in that it does not include all infants admitted to NICU, and so the generalizability to other study populations may be limited, however most infants transfused would be expected to appear in the NICUS data. Another limitation is that there may be errors in reporting in the gold standard Blood Watch data, particularly where red cells were released from the blood bank, returned, but not recorded as such, although such instances would be rare. These cases would falsely lower the sensitivity of the NICUS data.

## Conclusions

This study shows high consistency of reporting of transfusion, including quantity transfused, between the Blood Watch and NICUS databases. Although there is some under-enumeration, cases reported are likely to be true cases, as such, although the NICUS data may underestimate transfusion rates, it provides a valuable source of data for risk adjustment and analysis. The inclusion of quantity transfused in neonatal intensive care unit data provides reliable and useful additional information for research, compared with hospital data.

### Additional file

Additional file 1: Table S1. Population description. (DOCX 14 kb)

## Appendix

**Table 5:** Sensitivity and Specificity of transfusion reporting in hospital data compared with Blood Watch (hospital blood bank) data for infants in the Neonatal Intensive Care Units (NICUS) database

		Blood Watch Data	Hospital data	Sensitivity	Specificity	Positive predictive value	Negative predictive value
Total		637/ 3934 (16.2)	562/ 3934 (14.3)	83.7 (80.6, 86.5)	99.1 (98.7, 99.4)	94.8 (92.7, 96.5)	96.9 (96.3, 97.5)
Year	2007	154/ 905 (17.0)	134/ 905 (14.8)	84.4 (77.7, 89.8)	99.5 (98.6, 99.9)	97.0 (92.5, 99.2)	96.9 (95.4, 98.0)
	2008	172/ 980 (17.6)	146/ 980 (14.9)	82.0 (75.4, 87.4)	99.4 (98.6, 99.8)	96.6 (92.2, 98.9)	96.3 (94.8, 97.5)
	2009	157/ 992 (15.8)	134/ 992 (13.5)	81.5 (74.6, 87.3)	99.3 (98.4, 99.7)	95.5 (90.5, 98.3)	96.6 (95.2, 97.7)
	2010	154/ 1057 (14.6)	148/ 1057 (14.0)	87.0 (80.7, 91.9)	98.4 (97.4, 99.1)	90.5 (84.6, 94.7)	97.8 (96.6, 98.7)

## Abbreviations

NICU: Neonatal Intensive Care Unit
NICUS: Neonatal Intensive Care Units Data Collection
NSW: New South Wales
PPV: Positive predictive value
