# Supplementary material for: Comparison of neonatal red cell transfusion reporting in neonatal intensive care units with blood product issue data: a validation study
Source: BMC Pediatr. 2018 Feb 23;18:86. doi: 10.1186/s12887-018-1005-2 (PMC5824461; doi:10.1186/s12887-018-1005-2)
Supplement: Supplementary file 1 — Population description. (DOCX 14 kb) [file 12887_2018_1005_MOESM1_ESM.docx]

Additional file 1: Table S1: Population description

|  |  | **Births in tertiary hospitals** | **NICUS Data Collection** |
| --- | --- | --- | --- |
| Total |  | 95902 (100.0) | 3934 (100.0) |
| Gestational age | <=32 weeks | 3754 ( 3.9) | 2101 ( 53.4) |
|  | 33-36 weeks | 7012 ( 7.3) | 1107 ( 28.1) |
|  | 37+ weeks | 85136 ( 88.8) | 726 ( 18.5) |
| Multifetal pregnancy |  | 4510 ( 4.7) | 979 ( 24.9) |
| Apgar <7 at 5 minutes |  | 2190 ( 2.3) | 606 ( 15.4) |
| Private patient | Yes | 13166 ( 13.7) | 1110 ( 28.2) |
| SEIFA | Most disadvantaged | 16980 ( 17.7) | 966 ( 24.6) |
|  | 2 | 10714 ( 11.2) | 530 ( 13.5) |
|  | 3 | 21744 ( 22.7) | 552 ( 14.0) |
|  | 4 | 27705 ( 28.9) | 1039 ( 26.4) |
|  | Least disadvantaged | 17528 ( 18.3) | 772 ( 19.6) |
| Year | 2007 | 17230 ( 18.0) | 905 ( 23.0) |
|  | 2008 | 26014 ( 27.1) | 980 ( 24.9) |
|  | 2009 | 25961 ( 27.1) | 992 ( 25.2) |
|  | 2010 | 26697 ( 27.8) | 1057 ( 26.9) |

*NICUS= Neonatal Intensive Care Units’ Data Collection
